# Predictors of echocardiographic abnormalities in asymptomatic individuals with increased cardiothoracic ratio detected on chest radiography

**DOI:** 10.1016/j.ahjo.2026.100848

**Published:** 2026-07-30

**Authors:** Narongchai Wattanawongwon, Arjbordin Winijkul, Nithima Ratanasit, Kamol Udol

**Affiliations:** a Division of Cardiovascular and Metabolic Disease Prevention, Department of Preventive and Social Medicine, Faculty of Medicine Siriraj Hospital, Mahidol University, Bangkok, 10700, Thailand; b Division of Cardiology, Department of Medicine, Faculty of Medicine Siriraj Hospital, Mahidol University, Bangkok, 10700, Thailand

**Keywords:** Cardiomegaly, Increased CT ratio, Increased CTR, Increased cardiothoracic ratio, Asymptomatic

## Abstract

**Background:**

Cardiomegaly detected on chest radiography (CXR) is frequently encountered in clinical practice, even in asymptomatic individuals. However, its diagnostic value for identifying underlying abnormal cardiac structures in this population remains uncertain. This study investigated the predictors for the structural echocardiographic abnormalities among asymptomatic individuals with increased cardiothoracic ratio detected on chest radiography.

Methodology.

This is a cross-sectional study of asymptomatic adults who underwent routine health check-ups with increased cardiothoracic ratio (CTR) ≥ 0.5 on routine CXR between 2022 and 2025 at Siriraj Hospital, Bangkok, Thailand. A total of 452 participants with increased CTR on CXR who underwent echocardiography within six months were included. Individuals with known structural heart diseases were excluded. Predictive factors were evaluated, including medical history, abnormalities from physical examination and electrocardiogram (ECG).

**Results:**

Structural echocardiographic abnormalities were identified in 238 participants (52.7%). In the univariate analysis, age ≥ 65 years, hypertension, presence of a systolic murmur on physical examination, and ECG-detected left ventricular hypertrophy (LVH) were significantly associated with structural echocardiographic abnormalities. However, in the multivariable logistic regression analysis, only age ≥ 65 years, hypertension, and ECG-detected LVH remained independent predictors.

**Conclusion:**

Age ≥ 65 years, hypertension, and ECG-detected LVH were significant predictors of structural echocardiographic abnormalities among asymptomatic individuals undergoing routine health check-ups with cardiomegaly detected on chest radiography. These findings highlight the importance of integrating clinical assessment with electrocardiographic findings to guide referral decisions for echocardiographic evaluation, particularly in resource-limited settings.

Clinical trial number: Not applicable.

## 1 Introduction

Chest radiography (CXR) is a fundamental initial investigation with broad diagnostic applications for evaluating the lungs and mediastinal structures. It aids in diagnosing conditions, including pneumo-hemothorax, pulmonary nodules or infections, cardiomegaly, heart failure, and pericardial effusion. CXR is a non-invasive, cost-effective, and widely used diagnostic modality that is readily accessible throughout Thailand, including in rural and resource-limited settings. It remains a valuable tool for supporting differential diagnosis before undertaking further, more advanced diagnostic investigations.

Cardiomegaly is a common cardiac finding on CXR, with the reported rates ranging from 5 to 36% [1], [2], [3], [4], [5], [6] depending on study population and clinical context. Assessment of heart size on a CXR obtained in the posterior–anterior (PA) projection in the upright position is performed by calculating the cardiothoracic ratio (CTR). The CTR is defined as the ratio between the maximal transverse diameter of the cardiac silhouette and the maximal internal transverse diameter of the thoracic cavity, as measured on the PA upright CXR. A CTR >0.5 indicates cardiomegaly [7].

Cardiomegaly may result from various conditions, including left ventricular (LV) dilatation, LV hypertrophy (LVH), right ventricular (RV) dilatation, RV hypertrophy, left atrial (LA) dilatation, and right atrial (RA) dilatation. These abnormalities arise from diverse underlying etiologies and therefore warrant further evaluation. For individuals with suspected cardiac etiologies, including those with prior investigations suggestive of heart disease such as cardiomegaly detected on CXR, the American Society of Echocardiography (ASE) recommends transthoracic echocardiography for the evaluation of cardiac structure and function [8].

However, implementing this recommendation remains challenging across all healthcare settings in Thailand, particularly in rural areas. Although Thailand has been classified by the World Bank as an upper-middle-income country [9], substantial inequities in healthcare resources persist. Performing echocardiography for every asymptomatic individual with cardiomegaly detected during routine health check-ups may exceed the capacity of the Thai healthcare system. According to data from the Health Administration Division in 2021 [10], only 197 cardiologists were practicing in government hospitals nationwide, and 10 provincial hospitals had no cardiologist available. As a result, decisions regarding echocardiographic referral in routine clinical practice often rely on the clinical judgment of the attending cardiologist, based on clinical history, physical examination findings, and electrocardiographic (ECG) abnormalities. This reliance on individual clinical judgment may lead to variability and inconsistency in referral practices between clinicians.

Even when patients are referred for echocardiographic evaluation, this unequal distribution of healthcare resources adversely affects access to cardiovascular care, particularly for populations in rural areas where echocardiography is frequently unavailable. Consequently, patients often require referral from primary care hospitals to secondary or tertiary care hospitals within the same or neighboring provinces for further cardiac evaluation. Such referrals impose considerable financial, logistical, and social burdens on patients and their families.

Accordingly, there is a need to identify predictive factors that may help clinicians in Thailand and similar healthcare settings, particularly in low- and middle-income countries, stratify asymptomatic individuals with cardiomegaly on CXR who are most likely to benefit from further evaluation with echocardiography and should therefore be prioritized for referral. A study conducted in England demonstrated that asymptomatic individuals with a history of heart disease or established cardiovascular risk factors had a higher prevalence of echocardiographic abnormalities compared with those without such histories or risk profiles [11]. However, only one study from England has investigate predictive factors for echocardiographic abnormalities in asymptomatic individuals, and no comparable research has been conducted in Thailand, where healthcare systems, disease patterns, and resource availability may differ substantially.

Therefore, this study was designed as a cross-sectional investigation conducted at Siriraj Hospital, Thailand, to identify predictive factors for echocardiographic abnormalities among asymptomatic individuals with cardiomegaly detected on CXR. The objective of the study was to determine factors independently associated with structural echocardiographic abnormalities.

## 2 Material and methods

### 2.1 Study design

This retrospective cross-sectional study was conducted at Siriraj Hospital, Mahidol University, Thailand. The study population comprised asymptomatic individuals aged ≥18 years who underwent routine health check-ups between January 2022 and March 2025 and were found to have cardiomegaly on PA upright CXR, as determined by a radiologist and defined by a CTR ≥ 0.5. The study included only individuals who subsequently underwent transthoracic echocardiography. As part of routine clinical practice, transthoracic echocardiography was selectively performed at the discretion of the attending cardiologist based on individual clinical assessment.

Participants were excluded if they had any of the following: symptoms suggestive of cardiac disease (i.e., chest pain, dyspnea, palpitations, syncope); a history of structural heart disease, atrial fibrillation, ischemic heart disease, pulmonary hypertension or end-stage renal disease; a history of prior cardiac surgery; or peripheral edema on physical examination. Individuals with severe scoliosis that precluded adequate evaluation of the right heart border on CXR or with pleural effusion on CXR were also excluded.

The study protocol was approved by the Institutional Review Board of the Faculty of Medicine Siriraj Hospital (Certificate of Approval no. Si 337/2025) and included a waiver of informed consent.

### 2.2 Data collection

Baseline characteristics, coexisting diseases, and physical examination findings were retrieved from medical records. The most recent ECG data obtained within six months of the corresponding CXR were retrieved from the institutional database. Abnormal ECG findings were defined as the presence of LVH according to the Sokolow–Lyon voltages [12] or Cornell voltage criteria [13], left bundle branch block, right bundle branch block, poor R-wave progression or premature ventricular complex [14], [15]. All ECG findings were reviewed and interpreted by a board-certified cardiologist (NW).

The most recent transthoracic echocardiographic examination performed within six months of the corresponding chest radiograph was retrieved from the institutional database. All echocardiographic studies were conducted by cardiologists or certified sonographers and were subsequently reviewed and confirmed by an attending cardiologist as part of routine clinical practice. When required to ensure data completeness, additional echocardiographic measurements were performed by NW.

### 2.3 Outcome

Structural echocardiographic abnormalities were defined as the presence of ventricular systolic dysfunction, moderate-to-severe valvular heart disease, ventricular dilatation, or atrial dilatation. All definitions of structural echocardiographic abnormalities were based on established guideline recommendations from the ASE, including the Recommendations for Cardiac Chamber Quantification [16], Guidelines for the Echocardiographic Assessment of the Right Heart in Adults [17], Guidelines for the Noninvasive Evaluation of Native Valvular Regurgitation [18], and Guidelines for the Assessment of Aortic Stenosis [19]. Structural echocardiographic abnormalities were not mutually exclusive as an individual participant could have more than one abnormality.

### 2.4 Data analysis

We calculated that a sample size of 452 participants would be required to detect a difference in the prevalence of structural echocardiographic abnormalities between asymptomatic individuals with cardiovascular risk factors or a history of cardiac disease (53%) and those without (40%) [11] assuming a two-sided α of 0.05 and a statistical power of 80%.

Categorical variables were summarized as frequencies and percentages, and group comparisons were assessed using the chi-square test or Fisher's exact test, as appropriate. Continuous variables were presented as mean ± standard deviation if normally distributed, or as median with interquartile range (IQR) if non-normally distributed and compared using the independent *t*-test or Mann–Whitney *U* test, respectively. The normality of all continuous variables was assessed using both the Kolmogorov–Smirnov and Shapiro–Wilk tests.

Associations between predictive factors and structural echocardiographic abnormalities were evaluated using logistic regression, with results expressed as odds ratio (OR) and 95% confidence interval (CI). Variables with a p-value <0.20 in the univariate logistic regression analysis were subsequently included in the multivariable logistic regression analysis to identify independent predictors. A stepwise backward selection model was applied to derive the model, and a p-value of <0.05 was considered statistically significant. All statistical analyses were conducted using Stata version 19.0.

## 3 Results

### 3.1 Study participants

A total of 452 asymptomatic adults with cardiomegaly detected on routine CXR during health check-ups were included in the study. The median age was 64.1 years; nearly half of the participants were aged ≥65 years, and approximately three-quarters were female. Hypertension was present in approximately half of the participants. On physical examination, 12% had systolic murmurs, whereas fewer than 1% had diastolic murmurs. ECG-detected LVH was present in approximately 7% of participants. The median CTR was 0.53, and approximately 5% had a ratio ≥ 0.60. The baseline characteristics of the study participants are summarized in Table 1.

**Table 1:** Baseline characteristics of study participants (*n* = 452).

Characteristics	Total (n = 452)
Median age – year	64.1 (54.4–71.8)
Age ≥ 65 years	214 (47.4)
Female sex	344 (76.1)
Median BMI – kg/m^2^	25.8 (22.8–28.9)
Mean SBP – mmHg	138.0 ± 17.6
Mean DBP – mmHg	78.1 ± 12.2
Hypertension	243 (53.8)
Diabetes mellitus	110 (24.3)
Dyslipidemia	292 (64.6)
CKD stage III	28 (6.2)
Median eGFR (IQR) – (ml/min/1.73m^2^)	31.0 (33.0–52.0)
Obesity	260 (57.5)
History of ischemic stroke	30 (6.6)
Systolic murmur	54 (12.0)
Diastolic murmur	3 (0.7)
ECG: LVH	31 (6.9)
ECG: LBBB	3 (0.7)
ECG: RBBB	30 (6.6)
ECG: Poor R progression	50 (11.1)
ECG: PVC	29 (6.4)
CXR: CTR ≥ 0.6	23 (5.1)

Results are presented as number (percentage), mean ± standard deviation, or median (interquartile range). n; number of patients with available data, Body mass index; BMI, Bodyweight; BW, Cardiothoracic ratio; CTR, Chest radiography; CXR, Chronic kidney disease; CKD, Diastolic blood pressure; DBP, estimated glomerular filtration rate; eGFR, Interquartile range; IQR, Left bundle branch block; LBBB, Left ventricular hypertrophy; LVH, Premature ventricular complex; PVC, Right bundle branch block; RBBB, Systolic blood pressure; SBP.

### 3.2 Prevalence of structural echocardiographic abnormalities

Structural echocardiographic abnormalities were identified in 238 participants (52.7%). Among these, left ventricular systolic dysfunction was observed in 7 participants (1.5%), right ventricular systolic dysfunction in 4 participants (0.9%), and moderate-to-severe valvular heart disease in 21 participants (4.6%). The detailed distribution of structural echocardiographic abnormalities is presented in Table 2. Echocardiographic parameters not included in the definition of structural echocardiographic abnormalities are provided in Supplementary Table 1.

### 3.3 Comparison between participants with and without structural echocardiographic abnormalities

Participants with structural echocardiographic abnormalities were older and had higher proportions of age ≥ 65 years, hypertension, systolic murmur, and ECG-detected LVH than those without structural echocardiographic abnormalities. Diastolic murmurs were identified only among participants with structural echocardiographic abnormalities. Detailed comparisons between the groups are presented in Table 3.

**Table 2:** Structural echocardiographic abnormalities (n = 452).

Characteristics	Total (n = 452)
Structural echocardiographic abnormalities	238 (52.7)
LV dysfunction	7 (1.5)
RV dysfunction	4 (0.9)
Moderate-to-severe AS	5 (1.1)
Moderate-to-severe AR	3 (0.7)
Moderate-to-severe MS	1 (0.2)
Moderate-to-severe MR	6 (1.3)
Moderate-to-severe TR	6 (1.3)
LV dilatation	27 (6.0)
RV dilatation	59 (13.1)
LA dilatation	189 (41.8)
RA dilatation	56 (12.4)

Echocardiographic abnormalities were not mutually exclusive.

Results are presented as number (percentage).

n; number of patients with available data, Aortic regurgitation; AR, Aortic stenosis; AS, Left atrial; LA, Left ventricular; LV, Left ventricular hypertrophy; LVH, Mitral regurgitation; MR, Mitral stenosis; MS, Right atrial; RA, Right ventricular; RV, Tricuspid regurgitation; TR.

**Table 3:** Univariate logistic regression analysis of predictive factors associated with structural echocardiographic abnormalities.

Predicting factor	Structural echocardiographic abnormalities (n = 238)	No structural echocardiographic abnormalities (n = 214)	OR	95% CI	p-value
Age ≥ 65 years	138 (58.0)	76 (35.5)	2.51	1.71–3.67	<0.01
Male	60 (25.2)	48 (22.4)	1.17	0.75–1.80	0.49
Hypertension	150 (63.0)	93 (43.5)	2.22	1.52–3.23	<0.01
Diabetes	58 (24.4)	52 (24.3)	1.00	0.65–1.54	0.99
Dyslipidemia	156 (65.6)	136 (63.6)	1.09	0.74–1.61	0.66
CKD stage III	17 (7.1)	11 (5.14)	1.42	0.65–3.10	0.38
Obesity	129 (54.2)	131 (61.2)	0.75	0.52–1.09	0.13
History of ischemic stroke	20 (8.4)	10 (4.7)	1.87	0.85–4.09	0.12
Systolic murmur	38 (16.0)	16 (7.5)	2.25	1.27–4.35	<0.01
Diastolic murmur	3 (1.3)	0 (0.0)	1.00	–	–
ECG: LVH	26 (10.9)	5 (2.3)	5.13	1.93–13.60	<0.01
ECG: LBBB	2 (0.8)	1 (0.5)	1.81	0.16–20.05	0.63
ECG: RBBB	18 (7.6)	12 (5.6)	1.38	0.65–2.93	0.41
ECG: Poor R progression	30 (12.6)	20 (9.4)	1.40	0.77–2.55	0.27
ECG: PVC	17 (7.1)	12 (5.6)	1.30	0.60–2.78	0.51
CXR: CTR ≥ 0.6	13 (5.5)	10 (4.7)	1.18	0.51–2.75	0.70

Results are presented as number (percentage).

n; number of patients with available data, Cardiothoracic ratio; CTR, Chest radiography; CXR, Chronic kidney disease; CKD, Confidence interval; CI, Electrocardiogram; ECG, Left bundle branch block; LBBB, Left ventricular hypertrophy; LVH, Odd ratio; OR, Premature ventricular complex; PVC, Right bundle branch block; RBBB.

### 3.4 Univariate logistic regression analysis

In the univariate logistic regression analysis, significant predictors of structural echocardiographic abnormalities included age ≥ 65 years, hypertension, presence of a systolic murmur on physical examination, and ECG-detected LVH. The results of the univariate analyses are summarized in Table 3.

### 3.5 Multivariable logistic regression analysis

In the multivariable logistic regression analysis factors that remained independently associated with structural echocardiographic abnormalities included age ≥ 65 years (OR 1.96; 95% CI 1.31–2.96; p < 0.01), hypertension (OR 1.84; 95% CI 1.21–2.82; p < 0.01), and ECG-detected LVH (OR 4.15; 95% CI 1.52–11.35; p < 0.01). The results of the multivariable logistic regression analysis are presented in Table 4.

**Table 4:** Multivariable logistic regression analysis of predictive factors associated with structural echocardiographic abnormalities.

Predicting factor	OR	95% CI	p-value
Age ≥ 65 years	1.96	1.31–2.96	<0.01
Hypertension	1.84	1.21–2.82	<0.01
Obesity	0.67	0.45–1.02	0.06
History of ischemic stroke	1.52	0.66–0.35	0.32
Systolic murmur	1.49	0.77–2.88	0.24
ECG: LVH	4.15	1.52–11.35	<0.01

Confidence interval; CI, Electrocardiogram; ECG, Left ventricular hypertrophy; LVH, Odd ratio; OR.

## 4 Discussion

To our knowledge, this is among the first studies conducted in Thailand, an upper-middle-income country, to investigate predictive factors for structural echocardiographic abnormalities among asymptomatic individuals with cardiomegaly detected on CXR, with the aim of informing referral decisions, particularly in resource-limited settings. In our cohort, structural echocardiographic abnormalities were identified in 238 participants (52.7%), a proportion slightly lower than the prevalence reported in previous studies (61–88%) [6], [11], [20]. Variability across studies may reflect differences in study populations, particularly as our cohort consisted of asymptomatic individuals undergoing routine health check-ups without known underlying heart disease, the relatively low CTR observed in our cohort, the echocardiographic parameters included in composite outcomes, and evolving diagnostic criteria over time. In the present study, multivariable logistic regression analysis identified age ≥ 65 years, hypertension, and ECG-detected LVH as independent predictors of echocardiographic abnormalities.

Age ≥ 65 years emerged as an independent predictor, with an OR of 1.96 (95% CI, 1.31–2.96; p < 0.01) after multivariable logistic regression. Participants with structural echocardiographic abnormalities had a significantly higher median age (67.0 years) compared with those without abnormalities (60.0 years, p < 0.01). This finding is consistent with previous studies [21], [22], [23], which has demonstrated an age-related increase in the prevalence of cardiac abnormalities, including valvular degeneration [24], [25], and LAE [26], [27]. Taken together, these findings highlight the importance of considering age as a key determinant when interpreting cardiomegaly on CXR in asymptomatic populations.

Hypertension was also independently associated with echocardiographic abnormalities, with an OR of 1.84 (95% CI, 1.21–2.82; p < 0.01) after multivariable logistic regression. The prevalence of hypertension was significantly higher among participants with structural echocardiographic abnormalities (63.0%) compared with those without abnormalities (43.5%, p < 0.01). These findings are consistent with prior research [23], [28], underscoring the role of hypertension as a major contributor to concentric LVH [28], [29], [30] and LAE [31], [32]. Collectively, these findings emphasize the importance of accurate blood pressure assessment in clinical practice and highlight hypertension as a key determinant of the structural and functional cardiac changes underlying cardiomegaly.

ECG-detected LVH was another significant independent predictor, with an OR of 4.15 (95% CI, 1.52–11.35; p < 0.01) after multivariable logistic regression. The prevalence of ECG-detected LVH was significantly higher among participants with structural echocardiographic abnormalities (10.9%) compared with those without abnormalities (2.3%, p < 0.01). Our findings are consistent with previous studies demonstrating an association between LVH-detected by ECG and echocardiographic evidence of concentric LVH [33], [34]. However, ECG-detected LVH had a relatively low prevalence in our cohort (7%), which may partly reflect the known diagnostic performance of ECG criteria, characterized by high specificity (73%–98%) but low sensitivity (4%–67%) [35], [36], [37], [38]. This low sensitivity limits the utility of ECG-detected LVH as a stand-alone screening tool. Nevertheless, given the low cost and widespread availability of ECG, ECG-detected LVH may serve as a useful adjunct for risk stratification and prioritization of echocardiographic referral.

Although age ≥ 65 years, hypertension, and ECG-detected LVH are well-established risk factors for structural heart disease, the findings from our study emphasize the importance of these predictive factors even among asymptomatic individuals undergoing routine health check-ups with cardiomegaly (CTR ≥ 0.50) on CXR. Identification of these factors may provide a more objective approach to echocardiographic referral, rather than relying solely on clinical judgment, which may vary among clinicians or lead to indiscriminate referral of all individuals for echocardiography. Moreover, these findings may help clinicians prioritize echocardiographic referral among asymptomatic individuals with cardiomegaly on CXR particularly in settings where access to specialist referral and cardiac imaging is limited or where further investigations may impose substantial financial, logistical, and social burdens on patients and their families.

Several factors previously reported to be associated with structural echocardiographic abnormalities were not significant in our study. These included CKD stage III [39], [40], the presence of systolic [41], [42], [43] or diastolic murmurs [42], [43], ECG-detected left bundle branch block [44], [45], right bundle branch block [44], [46], poor R-wave progression [47], or premature ventricular complex [48], [49]. There are several possible explanations for these findings. First, the prevalence of some predictors in our cohort was relatively low, which may have limited the statistical power to demonstrate an independent association. Second, because our study outcome was defined as a composite of echocardiographic abnormalities, it is possible that certain predictors may be more strongly associated with specific abnormalities rather than with the overall composite outcome.

Although structural echocardiographic abnormalities were common in this study, most consisted of atrial enlargement, particularly LAE, whereas ventricular systolic dysfunction and moderate-to-severe valvular heart disease were relatively uncommon. LAE reflects structural remodeling in response to chronic pressure or volume overload [50]. In the absence of alternative causes, such as mitral valve disease, atrial fibrillation, bradycardia, or high-output states, LAE may reflect underlying left ventricular diastolic dysfunction [51], [52], particularly among older adults [53], [54] and individuals with hypertension [31], [55], [56], even in the absence of overt clinical manifestations [57], [58]. Nevertheless, its detection remains clinically relevant because LAE has been associated with adverse cardiovascular outcomes [59], [60], including atrial fibrillation [61], [62], ischemic stroke [63], [64], [65], [66], heart failure [67], [68], and mortality [63], [69]. However, specific recommendations for the management of isolated LAE, particularly strategies aimed directly at reversing left atrial remodeling in asymptomatic individuals, remain limited. Its detection should therefore primarily prompt identification and management of contributing conditions, together with optimization of modifiable cardiovascular risk factors [70], [71], particularly adequate blood pressure control in individuals with hypertension [72], [73], [74]. Although less immediately actionable than ventricular systolic dysfunction or moderate-to-severe valvular heart disease, LAE may still have clinical value by informing further evaluation and cardiovascular risk-factor management. Prospective studies are needed to determine whether this approach improves clinical outcomes in asymptomatic populations.

Another notable finding was the limited ability of CTR to distinguish between participants with and without structural echocardiographic abnormalities, despite its central role in defining the study population. A CTR > 0.60 was not significantly associated with structural echocardiographic abnormalities, in contrast to previous studies showing that higher CTR values are associated with greater specificity for structural heart disease [75], [76], [77]. This limited discrimination may partly reflect the restricted CTR range, as all participants had a CTR ≥ 0.50 and most had only mild radiographic cardiac enlargement (median 0.53; IQR 0.52–0.55). The exclusively asymptomatic nature of our cohort may also have contributed to the predominance of mildly increased CTR values. Consequently, only 5.1% of participants had a CTR > 0.60, limiting the statistical power to assess the association between more marked cardiomegaly and structural echocardiographic abnormalities. CTR measurements may also be influenced by noncardiac and technical factors, including body habitus [78], respiratory phase [79], and radiographic projection [80]. Consistent with our findings, a previous study using cardiac magnetic resonance imaging as the reference standard demonstrated that CTR values between 0.45 and 0.55 had limited sensitivity and specificity for detecting cardiac enlargement or dysfunction [75]. Although mildly increased CTR values may have greater sensitivity than more marked elevations, they have lower specificity for structural heart disease [75], [81]. Accordingly, mild CTR elevation should be interpreted cautiously in asymptomatic individuals and should not serve as the sole indication for echocardiography, particularly when access to echocardiography is limited. Rather, CTR should be considered alongside additional clinical and electrocardiographic factors to help prioritize echocardiographic referral, complementing rather than replacing clinical judgment.

Finally, although the ASE [8] recommends transthoracic echocardiography when structural cardiac abnormalities are suspected based on CXR or ECG, this recommendation may be difficult to implement in routine clinical practice in resource-limited settings, including Thailand. In such contexts, the findings of this study may help clinicians prioritize echocardiographic evaluation among asymptomatic individuals undergoing health check-ups with cardiomegaly detected on CXR who exhibit key predictive factors, namely age ≥ 65 years, hypertension, and ECG-detected LVH, thereby supporting more informed and efficient referral decisions where access to echocardiography is constrained. Moreover, this study represents an initial step toward future research in Thailand and similar healthcare contexts, particularly in low- and middle-income countries, on risk stratification among asymptomatic individuals with cardiomegaly detected on CXR and may ultimately help inform future healthcare policies and referral strategies in Thailand and similar healthcare settings.

### 4.1 Limitation

First, as this was a cross-sectional study, the assessment of exposures relied primarily on information extracted from medical records, which may not always be fully accurate. Such reliance could introduce non-differential misclassification bias, potentially biasing the results toward the null and obscuring true associations between predictive factors and echocardiographic abnormalities.

Second, as this was a retrospective cross-sectional study, the decision to perform echocardiography was based on clinical judgment and physician decision-making, which may have been influenced by patient characteristics, physical examination findings, electrocardiographic abnormalities, or perceived cardiovascular risk. As a result, the study population may represent a selected subgroup of patients considered by clinicians to be at higher risk of structural heart disease, potentially leading to an overestimation of the prevalence of echocardiographic abnormalities and limiting the generalizability of the findings.

Third, all echocardiograms in this study were performed as part of routine clinical care, making them inherently operator dependent [82], [83]. Although echocardiographic measurements are standardized according to ASE guidelines [16], [17], [18], [19], operator dependence may still have introduced variability in image acquisition and interpretation, potentially affecting the reliability of the findings.

Finally, this study was conducted at a single tertiary-care center in Thailand, and the study population consisted of asymptomatic individuals undergoing routine health check-ups within a specific healthcare context. Therefore, the findings may not be directly generalizable to other populations, healthcare systems, or settings with different patient characteristics, healthcare resources, and referral practices. Nevertheless, the findings may still be relevant to other low- and middle-income countries facing similar limitations in access to cardiovascular imaging and specialist care.

## 5 Conclusion

Age ≥ 65 years, hypertension, and ECG-detected LVH were identified as significant predictors of structural echocardiographic abnormalities among asymptomatic individuals undergoing health check-ups with cardiomegaly detected on CXR. These findings underscore the value of integrating clinical assessment with electrocardiography to support referral decisions for echocardiographic evaluation, particularly for higher-risk individuals in resource-limited settings. However, several predictors previously reported in the literature were not confirmed in this study, highlighting the need for cautious interpretation and further research to refine cost-effective diagnostic strategies in similar healthcare contexts.

## Abbreviations

ASE: American Society of Echocardiography
CI: Confident interval
CKD: Chronic kidney disease
CTR: Cardiothoracic ratio
CXR: Chest radiography
ECG: Electrocardiogram
IQR: Interquartile range
LA: Left atrial
LAE: Left atrial enlargement
LR: Likelihood ratios
LV: Left ventricular
LVH: Left ventricular hypertrophy
OR: Odd ratio
PA: Posterior–anterior
RA: Right atrial
RV: Right ventricular

## CRediT authorship contribution statement

**Narongchai Wattanawongwon:** Writing – review & editing, Writing – original draft, Visualization, Validation, Supervision, Software, Project administration, Methodology, Investigation, Funding acquisition, Formal analysis, Data curation, Conceptualization. **Arjbordin Winijkul:** Writing – review & editing, Visualization, Supervision, Conceptualization. **Nithima Ratanasit:** Writing – review & editing, Visualization, Validation, Supervision, Conceptualization. **Kamol Udol:** Writing – review & editing, Validation, Supervision, Conceptualization.

## Consent for publication

Not applicable.

## Ethics approval and consent to participate

The study protocol was approved by the Institutional Review Board of the Faculty of Medicine Siriraj Hospital (Certificate of Approval no. Si 337/2025) and included a waiver of informed consent.

## Declaration of Generative AI and AI-assisted technologies in the writing process

During the preparation of this manuscript, the authors used ChatGPT-5 (OpenAI) to assist in improving the clarity, readability, and language of the text. The authors take full responsibility for the content, analysis, and interpretation presented in this work.

## Funding

This research received no external financial or non-financial support.

## Declaration of competing interest

The authors declare that they have no competing interests.

## Data Availability

The datasets used and/or analysed during the current study are available from the corresponding author on reasonable request.

## Appendix A Supplementary data

Supplement Table 1 Echocardiographic parameters not included in the definition of structural echocardiographic abnormalities (n = 452).
