# Supplementary material for: Predictors of echocardiographic abnormalities in asymptomatic individuals with increased cardiothoracic ratio detected on chest radiography
Source: Am Heart J Plus. 2026 Jul 30;69:100848. doi: 10.1016/j.ahjo.2026.100848 (PMC13453017; doi:10.1016/j.ahjo.2026.100848)
Supplement: Supplementary file 1 [file mmc1.docx]

**Supplement Table 1. Echocardiographic parameters not included in the definition of structural echocardiographic abnormalities (n=452)**

|  | **Structural**  **echocardiographic abnormalities**  **(n=238)** | **No structural**  **echocardiographic abnormalities**  **(n=214)** | **p-value** |
| --- | --- | --- | --- |
| Median RWT | 0.51 (0.43-0.57) | 0.46 (0.40-0.53) | < 0.01 |
| Median LVMI – g/m^2^ | 99.0 (83.1-111.3) | 78.9 (65.4-91.9) | < 0.01 |
| Concentric LVH | 107 (45.0) | 35 (16.4) | < 0.01 |
| Eccentric LVH | 12 (5.0) | 1 (0.5) | < 0.01 |
| Mean LV diastolic diameter – cm | 4.3 ± 5.1 | 4.2 ± 4.4 | < 0.01 |
| Median LV diastolic diameter/BSA – cm/m^2^ | 2.6 (2.4-2.9) | 2.5 (2.3-2.7) | < 0.01 |
| Median LV systolic diameter – cm | 2.7 (2.4-3.0) | 2.6 (2.4-2.9) | 0.27 |
| Median LV systolic diameter/BSA – cm/m^2^ | 1.6 (1.4-1.9) | 1.6 (1.4-1.7) | < 0.01 |
| Mean LVEF – % | 67.8 (62.7-71.7) | 66.8 (62.1-70.0) | 0.05 |
| Median LA volume index – mL/m^2^ | 35.7 (34.1-39.8) | 27.6 (24.5-30.3) | < 0.01 |
| Median RV diameter basal – cm | 33.0 (30.0-37.0) | 31.0 (28.0-33.0) | < 0.01 |
| Median RV diameter basal index – cm/m^2^ | 19.8 (17.8-23.3) | 18.0 (16.0-19.8) | < 0.01 |
| Median RV diameter midventricular – cm | 23.8 (21.0-27.0) | 22.9 (20.0-25.0) | < 0.01 |
| Median RV diameter longitudinal – cm | 67.0 (61.0-72.2) | 64.5 (60.0-68.9) | < 0.01 |
| Median TAPSE – cm | 2.4 (2.1-2.6) | 2.3 (2.1-2.5) | < 0.01 |
| Median tissue Doppler S’ velocity – cm/s | 12.6 (11.5-14.1) | 12.2 (11.1-13.8) | 0.06 |
| Median RV longitudinal free wall strain  (three segment) – % | -34.8  ([-31.9]- [-36.7]) | -35.3  ([-31.4]- [-37.2]) | 0.40 |
| Median RA major dimension – cm | 4.7 (4.3-5.1) | 4.4 (4.1-4.7) | < 0.01 |
| Median minor dimension – cm | 3.0 (2.7-3.5) | 2.9 (2.6-3.1) | < 0.01 |
| Median RA area – cm^2^ | 13.0 (10.8-15.4) | 11.5 (9.9-13.0) | < 0.01 |

Results are presented as number (percentage), mean ± standard deviation, or median (interquartile range). n; number of patients with available data, Body surface area; BSA, Left atrial; LA, Left ventricular; LV, Left ventricular ejection fraction; LVEF, Left ventricular mass index; LVMI, Right atrial; RA, Relative wall thickness; RWT, Right ventricular; RV, Systolic; S’, Tricuspid annulus plane systolic exertion; TAPSE
